# The temporal and embodied structure of the mineness sphere: some phenomenological ideas to frame mental health

**DOI:** 10.3389/fpsyg.2024.1376665

**Published:** 2025-01-23

**Authors:** Camilo Sánchez Sánchez

**Affiliations:** ^1^Philosophy of Mental Health Unit, Department of Social Sciences & Humanities, Poznan University of Medical Sciences, Poznań, Poland; ^2^Faculty of Medicine, Institute of Human Genetics, National University of Colombia, Bogotá, Colombia

**Keywords:** phenomenological psychopathology, lived time, body movement, embodied cognition, mental health

## Abstract

This article highlights the import of two phenomenological variables: the implicit temporal and bodily movement experience. Then, I propose some ideas to build a framework for mental health. The proposal begins by critically considering Stanghellini’s conception of alterity, as he defines its relation through two conditions: reflexive self-awareness and “spoken word” dialogue. This conception prioritizes mental health work in the reflexive realm. In contrast, my conception prioritizes the pre-reflective realm of experience, in general, and focuses on the mineness sphere, in particular. This conception leads to consider two of Husserl’s phenomenological findings: first, awareness has a temporal structure, and second, temporal experience is constituted from the flow of consciousness. These findings are considered in terms of their constitutive import to subjectivity through the two variables. Next, I propose a general idea for a phenomenological framework of mental health work, integrating the two phenomenological variables with the concepts of alterity and ipseity. The psychotherapeutic approach known as “rhythmic relating” is considered to illustrate the key clinical role these two variables play, supporting the general aim. The conclusion presents the consequences of the proposal.

## Introduction

“*Nature is a principle of motion and change. (…) We must therefore see that we understand what motion is; for if it were unknown, nature too would be unknown*” (Aristotle, 1984, pp. 12–14).

Human life, identity, and dignity are forged in the life-long striving with what they do not (in principle) govern about themselves, others, and the environment. The result of the latter striving defines a mentally healthy (or not) individual or social group. Therefore, mental health is an achievement and a true social responsibility. Stanghellini (2017) approach is fine-tuned to this situated, contextual condition and re-synchronizes the individual to their social environment through dialectical and dialogical interaction and affective co-regulation.

Nonetheless, his proposal prioritizes the *spoken word* and *reflexive self-awareness* as therapeutic and diagnostic tools. Instead, this article proposes to balance the clinical encounter by prioritizing the *reafference* and dynamics of the living organism, that is, understood as the effect of the organism’s dynamics, behavior, and movement on itself including the experience (Vallortigara, 2021; Arikan et al., 2021; Jékely et al., 2021) like kinesthesia and proprioception, directly implicated in bodily movement. Contributing to advance the research project set forward by pioneers like Minkowski (1970) and Straus et al. (1969), and following Fuchs and Pallagrosi (2018, p. 297), the temporal experience is considered as one of the main axes of the psychopathological manifestations, while also attending to Maxine Sheets-Johnstone’s original developments of Husserl’s phenomenology, the experience of bodily movement and temporality lay bare (2014) as an embodied matrix.

This article contributes to a conceptual proposal for mental health grounded in the phenomenological pre-eminence of bodily movement and temporal experience as two main axes. The article is divided in seven parts: the first considers Stanghellini (2017) proposal, specifically regarding the individual relation with alterity, the second and third elaborate on the temporal structure of the minimal self and the constitutive process of temporal experience, correspondingly. The fourth elaborates on a therapeutic methodology, the fifth proposes a framework and the sixth explores some implications before concluding.

### Stanghellini’s model prioritizes reflexivity

Stanghellini’s proposal establishes *dialogical spoken word* and *reflexive self-awareness* as necessary conditions for the human encounter with alterity, which along with ipseity constitutes selfhood (Ricoeur, 1992). He identifies this encounter as defining mental health. The former is evidenced by the second principle grounding his model (PHD for *Phenomenology, hermeneutics, and psychodynamics*): “*Encourages the patient to reflect and express narratively his experience*” (Stanghellini, 2017).

This PHD model is developed in five steps, the first consists of the *unfolding of the patient’s life-world and its structure* (Stanghellini, 2017, pp. 118–121), a work mainly structured through the spoken word and reflexive self-awareness as the patient and the clinician produce a text based on the dialogical description and making sense of its own experience.

*“The product of unfolding is a text that reflects the phenomenal world, the world as it appears to the subject of experience… it is important to note that this process of unfolding is profoundly rooted in hearing—or even better: listening and dialoguing—and in the power of the spoken Word”* (Stanghellini, 2017).

The fundamental role of dialogue and reflexivity in Stanghellini’s proposal stems from his conception of the human being: *“…In brief: we are a dialogue with alterity. We encounter alterity in two main domains of our life: in ourselves, and in the external world. In the first case alterity is in the involuntary dimension of ourselves, our un-chosen ‘character’, including needs, desires, emotions, and habits. In the external world, alterity is encountered in the challenging otherness of the events and in the meetings with other persons that constellate our life”* (Stanghellini, 2017).

The latter fragment refers to the distinction between *alterity* and *ipseity.* The alterity refers to the sense of otherness stemming from what the individual cannot have direct agency over, like one’s involuntary nature (reactions, habits, moods, etc.), the others’ will, and the environmental forces. The ipseity refers to the sense of mineness stemming from what the individual has direct agency over: its individual resources.

*“Dialogue is a kind of ‘experience’: it is not merely a verbal exchange, an exchange of information; rather, dialogue lets something happen. What emerges in dialogue is neither mine nor yours and hence transcends the interlocutors’ subjective opinions”* (Stanghellini, 2017).

This fragment highlights that dialogue epistemologically “sharpens” the dialoguing parties and constitutes an inter-subjective sense through the inter-corporeal dynamics, that is, a “proto-dialogue” or “*proto-conversation*” (Stanghellini, 2017), which scaffolds the linguistic interaction. According to Stanghellini (2017), “*Dialogue is the main function of language*” because it serves understanding and mediates the human inner dialogue with alterity, which implies a *continuous process of becoming*, overcoming the immersion in mere now-moments and giving a way “*to the time of narration*.”^1^

Additionally, this linguistic relation with alterity entails self-awareness, reflexive or pre-reflexive depending on its features (Gallagher and Zahavi, 2008). This self-awareness comprises narrative skills and certain temporal experience (“time of narration”), therefore most probably it is an object-like or reflexive self-awareness.

Reflexive self-awareness is stated (Stanghellini, 2017) as a necessary condition for the constitution of individuality (“I”): “*The fullblown I only emerges once one perceives oneself as a You, when interpersonal dialogue turns into self- centered inner dialogue.*” The encounter with alterity (within) is linguistically mobilized and entails a reflexive self-awareness: “*Alterity comes into sight, materializes in the pleats of the world/text I have produced… A person cannot discern alterity within himself until he has made of himself an external reality by producing a text, and after reflecting upon it*” (Stanghellini, 2011).

Therefore, as claimed by this author, the encounter with alterity, linguistically mediated through dialogue and reflexive self-awareness, is associated with the full constitution of individuality. Another point highlighted by Stanghellini (2011) is that the structure of the patient’s text or personal narrative uncovers a discursive self-structure, which looks to integrate alterity narratively: “*Personal narratives are the result of the integration of alterity, revealed by the distantiation and autonomization of the text; in the discourse of the self* (Ricoeur, 1990)*. To note, the essential question is not to recover behind the text the lost intention of the author; rather, to unfold ‘in front of the text, the ‘world’ which it opens up and discloses’* (Ricoeur, 1981, p. 111).

Stanghellini’s values-based, person-centered model is grounded on the dialogical relation with alterity, which results from the development of the extended or narrative self in early socialization, along with the emergence of reflexive self-consciousness, narrativity, introspection, self-transcendence, and self-concept (Fuchs, 2010).

In his proposal, the latter serves as conceptual grounding to answer the main ontological questions: (i) What is a *human being*? (ii) What is *mental pathology*? (iii) What is *mental healthcare*? He conceives a human person as a being in dialogue with alterity; derived from this conception, mental pathology is the outcome of a crisis in that dialogue and, therefore, mental healthcare is the dialogical methodology through which the latter dialogue is re-enacted. The latter proposal is considered due to two reasons: first, to honor Stanghellini and its phenomenological model, and second, to criticize his limited conception of the relation with alterity, circumscribed to dialogue, as the argumentative ground for the proposal developed.

### The temporal structure of *minimal* and *extended* self

Fuchs (2010) grounds the extended self in pre-reflexive self-awareness as the individual basic sphere of first personal givenness of experience, following Gallagher and Zahavi, because the conceptual structure and skills require continuous and stable pre-reflective sense of mineness. This extended narrative self-entails conceptual competence regarding the sense of agency and self-story formulation.

From this *mineness* or *ipseity* (Fuchs, 2010) arises a basic form of selfhood or *minimal self.* This basic individual sphere entails the micro-structure of anticipations or protentions and retentions, through which functions *inner-time consciousness* or *immanent time* (Husserl, 2002). Also in this sphere are assembled, through a *passive synthesis* (Husserl, 2001), dispositions, potentialities, capacities, and habitualities, anchored in a continuous temporal structure and inter-subjective common sense.

As part of the bodily processes of movement and perception, the intentional function of consciousness presupposes a temporal synthesis: “*…f the continuity of temporal experience disintegrates, overarching meaningful units are no longer available, thereby creating temporal gaps in one’s stream of consciousness… this coheres with the hypothesis that a breakdown of temporality may be bound up with the breakdown of pre-reflexive self-awareness*” (Stanghellini and Ramella, 2015). Stanghellini highlights the importance of continuity, cohesion, and unity of temporal experience because these latter products of the temporal synthesis structure result in a sense of experience; therefore any synthetic disturbance can produce a disturbed experience.

There is an *implicit* and *explicit* temporal experience (Fuchs, 2019a). The former is pre-reflexively lived while the latter reflexively experienced. The implicit temporality is the ever-present undercurrent of experience, which presupposes the continuity of pre-reflexive self-awareness, resulting from the temporal blend of *presentation-retention-protention* or *transcendental synthesis* (Husserl, 2002).

Along with the continuous pre-reflexive self-awareness plays another factor, *conation*, which coordinates spontaneity, affective directedness, attention, and teleological behavior; these factors function as embodied vectors already assembled in the lived body. Constituting the lived body, there are habitual temporal sequences instantiated, like habits and actions; this implicit temporality of the lived body presupposes a whole-body dynamics, for example, kinetic-kinesthetic-proprioceptive-tactile-cenesthetic and body memory (Summa et al., 2012).

This basic distinction of temporal experiences is relevant in the inter-subjective realm (Fuchs, 2019b), as the “*relational order of processes which resonate/interact reciprocally*.” The implicit inter-subjective temporality is grounded on inter-corporeality, which entails ongoing *attunement* and *resonance* and is associated with synchronic movement micro-dynamics; similar conceptualizations have been proposed by Minkowski’s *lived synchronism* (1970) and Fuchs’ *basal contemporality* (2019) to point to the inter-corporeal ground of the social sense. By contrast, the explicit inter-subjective temporality is associated with desynchronization.

A fundamental phenomenological finding is that the temporal experience derives from the *absolute flow of consciousness* (Husserl, 2002). Following Husserl, it is constituted through the *primal synthesis*. This constitutive process is elaborated in the next part.

### The *absolute flow of consciousness* and temporal experience

Husserl’s concept of absolute flow (Husserl, 2002) refers to an *unchangeable form of presence* (“*nunc stans*”) of a continuous, ever-present, and ever-changing phenomenal flow. The consciousness flows as *absolute subjectivity* in the absence of any temporal objectivity (Husserl, 2002), having as primal source a bodily enriched streams of inter-dependent phenomenal consciousness. This multiplicity of content converges into *one* subjective flow of consciousness and the *unity* depends on Husserl (2002): (i) *transformation law* and (ii) *intentionality*. The transformation law points to the Heraclitean nature of this primal consciousness as *flowing change or movement* characterized by Husserl through three *identity traits*: (a) *familiar form or way of the* “*now*,” (b) *homogenous flowing manner*, and (c) *tempo identity*. The complementary function of *transverse* and *longitudinal intentionality* constitutes the immanent temporality and the actuality phases, correspondingly.

The longitudinal intentionality constitutes a pre-phenomenal ordering of the flow phases, that is, from a necessary actuality phase (“now”) derives pre-actual and post-actual phases (Husserl, 2002). The transverse intentionality constitutes temporal objectivities characterized by *duration* and *permanence* (Husserl, 2002). Regarding the transformation law and the three *identity* traits, the continuous flow of subjectivity is the proto-temporal consciousness and its *flowing manner* and *synchronicity* correspond to the main characterization of *unity* and *identity* of the primal flow of consciousness, suggesting some questions: *Does the identity of the flow convey an individual identity*? The form is the ever-changing flow, *oriented by the individual body. Does the remaining two identity traits define individuality*? for example, an individual pattern or “rhythm” of consciousness.^2^

From the flow of consciousness, the intentional function constitutes or synthesizes any consciousness of *unity* or *identity* (Husserl, 2002). This phenomenological characterization enlightens the basic Heraclitean character of human consciousness, that is, *movement or flow*, and also hints about its disturbances, that is, always entailing a *stillness* or immobility of such flowing nature. The disturbance always denies the characteristic movement or flow of consciousness pathologically, affecting its unity and identity, in terms of its three identity traits. But the disturbance can also arise from any pathological alteration in the diversity of constitutive processes, for example, the inter-dependence structure of the (pre-phenomenal) actuality phases and/or continuous synthesis, association, and homogeneity-based functions (Husserl, 1980; Husserl, 2001).

The disturbance of these constitutive processes can be illustrated through the first trait of the *transformation law*. The “*now*” entails a structure that seems ontogenetically shaped, but how does the bodily flow of reafference and sensory feedback contribute to the structure of the “*now*”? Depending on the conditions of the reafference’s flow pattern, the individual adjusts or tunes its presence in the “*now*,” for example, a teenage girl just beginning her sexual life, due to certain circumstances with her boyfriend, begins building (for some months) a fear toward sexual intercourse when confronted again with a situation potentially sexual; every move and gesture seems to trigger again the retention of the episode that left her openly vulnerable, and even though she is bodily drawn into this pleasure, her reafference patterns rapidly flow from the agitation of falling to an escape into certain passivity of shutting down or inhibition blockage pattern. This latter bodily flow tends to an overall contraction or stiffed stillness depending on the particular environmental (intersubjective) challenge. Therefore, according to the challenges (alterity) or threats experienced by the individual, the structure or presence in the “now” is tuned through the bodily reafference patterns, which define the individual movement affordances (ipseity). Based on the latter, it becomes clear how the whole pattern of self-affection, through the reafference and sensory feedback, brings individual modifications to the temporal and movement experience and therefore to the structure of the “now.”

These patterns of avoidance involve whole-body reactions of tension and clenching, for example, eyes, hands, toes, and teeth. This constriction of the structure of bodily sense entails a collapse of the spatial and temporal experience, that is, the same way as the extremities constrict in their *egocentric spatial frame* (Gallagher, 2006) or bodily subjective axis, the whole body tends to lose its egocentric spatial support or *disappear in space.*^3^ Similarly, the temporal experience becomes distorted by a progressive hyper-retentionality through which the individual tries to modify the flow of consciousness, resulting in a protentional speed-up as trade-off. The threatening event modifies the temporal dynamics by transiently denying sprouting protentions and the whole body tends to lose its ontological duration and continuity.

The example enlightens a cardinality in the structure of body sense, which shapes the temporal and movement experience. Humans have the most vulnerable points in the frontal torso, for example, heart, lungs, digestive system, and main arteries as an evolutionary phylogenetical heritage to cope with through protention and bodily movement and their main (upright posture, Straus, 1952) locomotion is also structured upon the same cardinality: Humans advance by moving forward. This cardinality serves not only how the movement affordances are structured but also simultaneously how the temporal experience unfolds. As most animated living beings structure their movement according to their caudal-ventral anatomical structure, thicker bones cover the back while ventral vital organs must be protected through perceptual and movement functions. These adaptive mechanics of locomotion enable them to foresee any coming threat, but also as the vital and organic bodily movement leaves behind the path already traveled looking to what is coming and yet to come, the temporal experience flows in the same manner through the retention and protention dynamics.

This cardinality and structure of body sense portrays an organic bodily instantiation of ipseity and alterity, the latter being some naturally inherited vulnerabilities and the former being the individual’s vital forces to cope with those vulnerabilities, both shaped through phylogenetical and ontogenetical pathways. Individuals also cope with all-natural forces circumstantially, becoming opposition to its way, for example, the radical bodily experience of alterity is dealing with one’s weight under gravity conditions throughout life. Conducting one’s own body is an early encounter with alterity, which soon becomes transparent and then is brought back to the fore of consciousness during the older years. These continuous challenges lay bare the individual’s alterity, but also can prompt self-awareness and self-confidence or long-term sense of agency (Marcel, 2003) only when the living organism is willing to accept the encounter and willfully cope with its alterity, correspondingly.

The latter illustrates how the “now” is structured or tuned through the reafference and sensory feedback flow patterns, which impact how the temporal and movement experiences are structured. Thus, individual attunement in the “now” depends on phylogenetic and ontogenetic pathways. According to the modifications of the bodily flowing patterns (reafference and sensory feedback), the hypothetical (body) sense structure could also be modified via the kinesthetic memory, modifying the structure of available affordances, that is, the actual movement possibilities. This implies a modification of the whole-bodily pattern of carrying one’s weight, for example, popular sayings refer to *being on your toes* or (being) *on your back foot*, as a way of referring to a disposition prone to movement or to give space to others (ready to respond to them) correspondingly. The particular weight distribution continually adjusts the structure of affordances and hints toward the retention-protention dynamics, because if someone is *on their toes*, their temporal experience landscape is leaning toward and highly acute in protentionality. Therefore, the individual presence in the “now” alters its structure of affordances and whole-body weight distribution and vice versa, for example, after a traumatic experience individuals could live through their own time as *going backwards*, avoiding facing the upcoming events and dissociating from their protention. The latter metaphor conveys the individual existential position in the “now,” which could be as if one is going on a train in a seat facing backwards to the movement direction, so the landscapes are seen through the window when they are fading in the distance, that is, they are seen in the retentional range of experience to avoid facing them protentionally.^4^

Based on the latter, it becomes clear how the whole pattern of self-affection, through the reafference and sensory feedback, brings individual modifications to the temporal and movement experience and therefore to the structure of the “now.” The remaining two Husserlian conditions or identity traits point to the ontological foundation of individuality. Due to the space limitation, only this first feature of the constitutive process is elaborated. The next part proposes a framework for mental healthcare.

## Discussion: toward an integrative framework

The dialogical spoken word and reflexive self-awareness are fundamental in psychotherapy in general. Nonetheless, their priority has led to dismissing a prior “proto-dialogical” landscape of the inter-subjective encounter and the therapeutic potential richness it offers. The present proposal prioritizes the latter as the focus of the therapeutic intervention, based on the individual relation with alterity as primarily^5^ encountered in one’s and the other’s bodily movement, that is, through coordinating the bodily movement against natural forces like gravity.

The primal presence of the stream of consciousness is from where the experience of unity and identity is constituted, that is, the experience of oneself, the other, and the environment through the temporal and passive synthesis. The mineness sphere is structured through pre-reflexive self-awareness and immanent temporality and the proposal is to study mental health disturbances through the temporal constitutive process “lens”; then the pre-reflexive self-awareness sphere is the focus because even the dialogical and reflexive aspects of the therapeutic intervention can benefit from the proto-dialogical therapeutic work.

Parnas and Henriksen (2014) propose to identify an individual’s subjectivity (ontological) structure as the dynamical relation with alterity, that is, the affective differentiation and coalescing during the intersubjective encounter. This circular pre-reflective flow from identity (ipseity) to otherness (alterity) grounds the individual subjectivity and reflexivity. This hypothesis (Parnas and Henriksen, 2014) is inspired by Ey (1973) and Kimura (1992) notion of *áïda* or *in-between-ness,* explaining the individual co-constitution through circular dynamics of differentiation and coalescing: “…*the notion of ‘in-between’ is not to be understood as a static or spatial distance between two different things, but rather as a dynamic and generative movement that differentiates one thing from the other… it seems to be the dynamic nature of the ‘in-betweens’ that is disturbed in schizophrenia, in that, the otherness of subjectivity has become concretized or thickened”* (Stephensen and Parnas, 2017).Kimura’s proposal conceives the co-constitution of subjectivity and alterity (the other and the world) as a generative movement from which the individual, its world and its relation with others is constituted. *“In other words, the precondition for being able to recognize oneself, on an implicit or immediate level, consists in the interplay of integration and differentiation. Alterity is thus a constitutive feature of subjectivity, and in schizophrenia this alterity remains exposed or non-integrated, and one becomes Other to oneself in a concrete manner”* (Stephensen and Parnas, 2017). Following Kimura, the proposal from Parnas and Stephensen also focuses on the pre-reflexive realm to track down major psychopathological manifestations, but how does the generative movement between differentiation and coalescing constitute subjectivity and alterity? This question will have to be addressed in the another upcoming study.

The latter inspires the present proposed framework. Alterity continuously challenges animated life, this striving brings consequences, individual and social, lessening or amplifying animate ways of living. This life-long encounter with alterity, which carves and shapes individual identity, entails a flow of experience between two poles: *subjectivity* and *otherness*. These poles embody ipseity and alterity correspondingly.^6^ The subjectivity pole can be instantiated through the flinching or startle reflex (Sheets-Johnstone, 2016) as a radical (experience of) individuation from situated alterity that is, this pattern of bodily sense (reaction) instantiates when someone is attacked or suddenly gets into cold water. The otherness pole can be instantiated through the lordosis reflex, as a radical we-experience (coalescing) or fusion with situated alterity, for example, this pattern of bodily sense (reflex) instantiates in copulation; this pole is not necessarily understood as affective, rather refers to a general broad kind of experience in which individuality “dissolves” into a social phenomenon.

Individuals develop sense-making routines shaped by phylogenetic and ontogenetic conditions (De Haan, 2015), for example, affable gestures when presenting a disagreement with the social group to probe its probable response. These latter bodily patterns become habitual temporal structures of behavior, contributing to attaining a sense of experience, which becomes a repertoire according to the individual’s history, species, and genre. These sense-making routines unfold coherently with the reafferent flowing; that is why there is an individual rhythm according to which these routines are displayed, for example, how often an individual looks or speaks to another, initiating a conversation. This individual rhythm contributes to the unfolding of experience and the sense constituted.

“…*an originary and even proper elucidation of how time-consciousness is possible lies not in the examination of something external to us, like a melody, but in the very nature of our being the animate organisms we are, that is in the very nature of self-movement*” (Sheets-Johnstone, 2014).

These constitutive flows between ipseity and alterity instantiates through bodily movement and temporal experience. This flow takes place in the inter-corporeal dynamics (movement and action) and micro-dynamics (gaze, gesture, posture), leading to a potentially synchronic, fine-tuned social interaction, sometimes unveiling a shared temporal experience.

The next part presents a psychotherapeutic methodology illustrating the key role of bodily movement and temporal experience in mental health disturbance.

### A clinical approach toward an integrative pathway

Recently there is an explosion of research on phenomenological psychiatry and mental healthcare rooted in phenomenology. This article proposes a conceptual framework structured upon two phenomenological variables to integrate the theoretical, experimental, and clinical work. These are main variables for psychotherapeutic methodologies being applied, like *rhythmic relating* (Stuart et al., 2022) approach where elements from dance movement, improvisational music, play and musical interaction therapies are assembled with the influence of the Communicative Musicality model. This approach fine*-*tunes bodily movement and temporal experience to enable reciprocal regulation and transformative interaction. The aim of this section is to highlight the clinical relevance of these two variables in the rhythmic relating approach to autism spectrum disorders (ASD), as it entails a timing impairment in social interactions (Rochat et al., 2013; Di Cesare et al., 2017) or fundamental social asynchrony (Casartelli et al., 2020).

The power of this approach lies in the commitment to therapeutically work on the rich phenomenological sense of the body including its constitutive elements, that is, touch and kinesthesia (Husserl, 1989). This original background is mobilized and “massaged” through rhythmic interactions, frequently involving bodily movement. This creative approach is broad in terms of the variety of therapeutic tools, and yet focused on the proposed bodily variables or axis, constantly exercised through the dynamical flowing pattern of reafference.

The rhythmic relating methodology exercises flowing patterns through bodily playful interaction, engaging the elements of the flow of consciousness, the constituted experience of time, and its corresponding intentional arc (Merleau-Ponty, 1962), invigorating and developing healthier and more adaptive patterns. These interactions regulated by primary intersubjectivity (Trevarthen, 1998; Trevarthen and Delafield-Butt, 2017) and *communicative musicality* (Malloch and Trevarthen, 2009) are also referred as kinetic melody (Luria, 1973) or dance unfolding (Stern, 2010).

This paradigm of social interaction highlights timing as determinant in primary intersubjectivity, which builds upon the shared intentionality for action. The intersubjective pattern is inherently rhythmical, as humans perceive, move, and interact in rhythmic ways, that is illustrated by the interaction between the neural structuring of bodily movement and the organism’s cyclic temporality (Osborne, 2009).

This methodology promotes a rhythmic social interaction finely tuned through coordinated bodily movement in assembly with others, entailing a finely tuned reciprocal protention. Another aspect of the bodily interaction is synthetized by Luria’s *kinetic melody,* referring to a synthetized kinesthetic integral structure activating a dynamic stereotype of automatically interchangeable elements (Sheets-Johnstone, 2012). The kinesthetic and kinetic melodies (Luria, 1966; Luria, 1973) are understood through one’s signature or washing one’s teeth: These bodily dynamics (Sheets-Johnstone, 2014) unfold pre-reflectively through well-known constituted patterns of reafference, including kinesthetic, kinetic, proprioceptive, tactile, auditory, and visual feedback, which draw immediate attention when broken. These habitual flowing patterns carry a characteristic sense of one’s individuality, termed by Husserl as absolute subjectivity (Husserl, 2002).

A key phenomenological and neurological aspect of the unfolding of kinesthetic and kinetic melodies is that they are constituted within a temporal experience. The unity of kinesthetic structures is partially dependent on their rhythm (Luria, 1966; Luria, 1973); they can be primed by a single stimulus to instantiate the whole continuum which specifies a rhythm, for example, a kiss unfolds certain kinesthetic and kinetic rhythmic pattern. This type of individual bodily dynamics is intimately related to the (individual) physiology and cyclic temporality of a living organism for example, neurotransmitter/hormonal activity and energy expenditure (Fuchs, 2021). Complementarily, the animated (Sheets-Johnstone, 2010) coordination of affect with goal-directed behavior is structured upon kinesthetic patterns. Thus, these constitutive bodily patterns serve intersubjective interaction through the rhythm and temporal experience constituted upon them, enabling individuals to share and bond (Feldman Barrett, 2017), as the following therapeutic exercise portrays.

“*While running, the client* (patient) *looks briefly in the direction of practitioner* (therapist) *a couple of times, and when getting closer to the corner he looks at the practitioner’s hand through the whole ‘aaand…. Stop!’ moment. The client smiles as he recognizes that the practitioner is following his movements, and right after the ‘Stop!’ pause, the client makes a shorter run before the next ‘aaand…. Stop!’ event. This is repeated a couple of times, and to ensure the continued shared interest, the practitioner starts to act a bit clumsy, and makes the ‘Stop!’ a little too late (or later too early) while showing a confused, yet smiling face (an appropriate, engaged level of humour)*” (Stuart et al., 2022).

This fragment portrays a therapeutic approach combining rhythmic relating and improvisational music therapy, which focuses on reinforcing the basic temporal cohesion of experience through the intentional arc, which refers to the intentional processes running over the temporal synthesis of protention–presentation–retention (Merleau-Ponty, 1962): going over and over the “aaand…. Stop!” The therapeutic study focuses on synchronically recruiting the coordinated reafferent flow to exercise the build-up of affective tension and relief, on which the dynamics unfold and scaffold the social interaction through synchronic bodily movement.

*Kinesthetic flow* and *rhythm* are corresponding key notions to movement and temporality. They structure the social interaction and play a central role for protention; for example, similar to musicians, people build stable relations by learning to anticipate each other’s behavior (Manders et al., 2021). As these psychotherapists state (Stuart et al., 2022), social motor synchrony “*relies on predictions generated within the rhythmic flow of interaction and, in turn, enables the furthering of those rhythms.*”

In a therapeutic session (Stuart et al., 2022), the psychotherapist looks to engage the patient rhythmically through bodily movement or sounds, for example, building up momentum upon the patient grabbing a toy train through repetitive iterations of the same rhythmical prompt: “*choo-choo*.” Following the patient’s request and intensity, every time louder and faster, finally displaying a more tumultuous steam sound effect along a 4/4 beat with their hands drumming on the floor. This dynamic actually peaks when, after the build-up, the patient crashes the train with a loud turbulency sound effect from the therapist. Afterwards, patient and therapist smile and make eye contact. The patient withdraws for a moment and then reorients, uprights the train, and urges the therapist to continue, followed by a rhythmic engagement, which the therapist regulates in its intensity (volume and energy) through three categories: “louder,” “quieter,” and “quiet.” This shared rhythmic experience enables the patient to exercise agency in social interactions and regulate its wellbeing.

The kinesthetic flow along with the complementary reafference, for example, the proprioceptive and tactile patterns, complemented along the kinetic, visual, and auditory assembled feedback, contribute to building a distinct sense of individuality because they shape and articulate the basic patterns of animation, for example, most common intentional arcs structuring reactions and all-round behavior in terms of rhythmic bodily movement. The kinesthetic and reafferent flow integrates to the physiological rhythmic pattern of the living organism, affecting and being affected by hormonal/neurotransmitter activity, blood pressure, breathing rate, etc. The kinesthetic and reafferent flow included in the rhythmic patterns move the living organism from the time in the womb throughout the vital developmental process; some of these rhythmic patterns become habitual as *kinesthetic memories* (Sheets-Johnstone, 2010), for example, the kinetic melody of a sequence of salsa steps learned since teenage years and cued by certain gesture or bodily movement.

Based on the latter, a working hypothesis is introduced. There is a basic sense of individuality constituted by a whole-body habitual pattern of reafferent dynamics. Following Brian O’Shaughnessy’s conception of *body image* (O’Shaughnessy, 1998) understood as a primitive body structure grounded on kinesthetic–tactile–proprioceptive dynamics and memory, and Maxine Sheets-Johnstone’s *tactile–kinesthetic body* (Sheets-Johnstone, 2010), a sense of body structure is proposed as ground of individuality and selfhood. The cohesive sense of body constituted through the long-term flowing patterns of reafferent dynamics, in reciprocal constraint with the musculoskeletal conditions of the articulated body, hierarchically define a structure of possible movements, that is, a sense of body structure in terms of rhythmical movement. The kinesthetic and reafferent flowing patterns, proven adaptive and promoting the individual’s life, become habitual and phenomenologically “transparent” or recessive. This individual structure becomes habitual through the consolidation of kinesthetic memory (Sheets-Johnstone, 2010).

Based on the above, the experience of body movement and time are deeply intertwined, as they structure upon the stream of consciousness and the reafference dynamic, through which this therapeutic methodology establishes the rhythmic interactions. Thus, the structure of the sense of body can be articulated coherently to the phenomenological approach and this therapeutic methodology through conceiving the body as regulated by a rhythmicity expressed in movement, emotion, and purposeful behavior. Koch and colleagues also suggest a whole-body pattern expressing an integral stream; this proposed hypothetical structure ontologically grounds individuality in articulation with the minimal and extended (or narrative) self, epistemologically enlightening new approaches to psychopathological manifestations and mental health in general. This hypothesis enables advantages in the clinical realm regarding diagnosis and therapeutic approach, which will be considered in the last section.

This proposal shares conceptual ground with the experimental study in neurosciences and computational approaches to mental health research. The study in neuroscience associates the activity of the default mode network (DMN) to instances of the temporal experience unfolding (Tran The et al., 2022), which actually corresponds to the mineness or self-affection sphere synchronizing the pre-reflexive reafferent bodily dynamics (kinesthesia, cenesthesia, etc.), along with the cyclic temporality and the physiological pattern of energy expenditure supporting the reflexive activity entailed.

Tran The et al. (2022) articulate the neuroscientific findings with Freud’s account of psychotic symptoms, understanding them as a compensatory binding function of the disturbed experience and selfhood in schizophrenia. These neuroscientific findings of the DMN articulate the fundamental role of the temporal experience with the body and self-referential content of experience. This content corresponds to the complex bodily dynamic of the mineness sphere: kinesthesia, proprioception, cenesthesia, interoception, and touch, which are synchronized in the sense of the body experience. The body dynamic unfolds in sharply rhythmical patterns, which ground the sense of individuality and selfhood experience. In this sense, schizophrenia patients suffer a disturbed sense of bodily experience.

The abnormal hyperactivity of the DMN (Tran The et al., 2022) in the precuneus, prefrontal cortex, and parietal areas is associated with episodic and autobiographical memory, protentional capacity, and the epistemic skill to know about others body states, all of which directly ground the two bodily axis or phenomenological variables proposed as key pillars for the work in mental health. This proposal can be articulated with the *free-energy principle*^7^ (FEP) and computational approaches by formalizing the temporal experience through the Bayesian approach, Markov decision processes, and Forney-style factor graphs (Bogota and Zakaria, 2023), which ground *active inference*. The latter approach simulates the temporal dynamics conceptualized through phenomenology (Albarracin et al., 2022) to experimentally assess specific problems relative to this subjective experience. Accordingly, this proposal could be experimentally tested through these simulations approach, bringing potential new knowledge to the arising problems.

The following part synthetizes the limitations and advantages of the proposal as part of the concluding remarks.

## Conclusion

This proposal contributes to phenomenological psychiatry by articulating some ideas to work out a conceptual framework, highlighting two phenomenological variables as key pillars and axes for the study in mental health. The main limitation of this proposal is the lack of a bridge between the concepts and the experimental tests or clinical applications. The forthcoming experimental research study and clinical training will gradually overcome this limitation.

There are also some advantages of this proposal, simplified in the following points:

(a) The conceptual study identifies key realms and variables for psychopathology and psychotherapy and it is a complementary methodology to the research in neuroscience, psychiatry, and pharmacology because the accurate conceptualization enables solid associations within the experimental and clinical findings.
(b) The phenomenological study offers a strong and economic epistemological framework, honed to explore the implicit realm. This study situates the clinical encounter in the encounter with alterity, which entails the involvement of relatives, a re-educational process, and the use of pharmaceutical aids as part of the therapeutic strategy.
(c) New therapeutic approaches flourish due to the phenomenological conception of the body, as it stimulates free and creative body work. This proposal promotes a movement-based and rhythmically structured body work for diagnostic and therapeutic purposes.
(d) This proposal promotes embodied subjective awareness and develops individual authenticity and freedom through a re-education process based on habits and framed by a mental health continuum between education and clinical work, that is, between healthy and unhealthy. It also exercises agency through bodily movement, situated social re-synchronization, and building a close social group around the individual, willing to reciprocally commit to the therapeutic strategy and goals of the recovery process. The intervention brings awareness of the social responsibility and accountability entailed by mental health.
(e) This proposal promotes social cohesion by working to integrate the affected individual through an educational process of the immediate social group, along the therapeutic process. The latter builds social accountability and awareness about how mental health is a building block of the social, economic, and political realms.

The above highlights the relevance of studying these constitutive processes of experience, addressing them through conceptual and experimental study, and looking to learn about their import to the constitution of subjectivity and individuality. This study offers a solid perspective and tools for mental healthcare in general. Husserl’s phenomenological findings and conceptual developments offer the tools and landscape for an efficient and simpler psychopathology and psychotherapy. The resulting criteria are the lack of continuous rhythmical flow between the two foundations (alterity and ipseity) of subjectivity or a *stillness* in the stream of consciousness signals a lack of mental health.

This continuous and rhythmical flow associated with mental health is vitally exercised throughout an individual’s life, as part of the social interaction but also when self-invested into an activity or routine, for example, in a romantic relationship lovers spend themselves to the fullest in communion and reserve themselves for individuation when each other’s alterity threatens the wellbeing in communion. Individuals’ life is spent through ongoing cycles of investing the vital forces in developing capacities and skills to cope with adversities and limitations met in the environmental challenges or on the other’s will but certainly also in the individual’s shortcomings. This proposal looks to frame mental healthcare by this vital cycle dynamic and promote the design of new therapeutic strategies to support affected individuals by reinforcing this dynamic in an educated and responsible way.

## Footnotes

^1^This conception of a Heraclitean ever-changing human nature is enlightening and accurate. Classically understood a paradigmatic example of this definition is *movement* and *time*, unsurprising as they are necessary assumptions (along with *space*) of the concept of change and transformation.

^2^Alluding to the temporal traits of the flow of consciousness.

^3^This whole bodily pattern constitutes affective conditions such as anguish.

^4^Referring to animals capable of self-initiated movement.

^5^This is an ontological priority, which entails those individual narratives stemming from individual embodied qualitative dynamics (Delafield-Butt and Trevarthen, 2015). Notwithstanding, this issue is not the goal of this study.

^6^These conceptual links are much more complex. Individuals can reciprocally bond through and to either poles of selfhood: ipseity or alterity. How a *we-experience* or other types of bonds result and their prompters of a potential pathological or healthy condition require a much more in-depth analysis.

^7^This principle proposes to conceive the brain as a system to maintain balance and predictability. This principle can be related to (i) the temporal structure of consciousness which through the protentional-retentional dynamics contributes to cognitive predictability; (ii) the DMN hypothetic function of balancing between the internal and external stimuli (Tran The et al., 2022); and (iii) to alterity and ipseity as a potential parallel distinction to homeostasis and allostasis. Homeostasis is a balance based on preserving conditions and allostasis a balance based on change. Thus, there is an organic balance to these two aspects of selfhood. Due to space limitations, these relations cannot be elaborated further.
